# Synthetic Biology-Driven Microbial Production of Resveratrol: Advances and Perspectives

**DOI:** 10.3389/fbioe.2022.833920

**Published:** 2022-01-20

**Authors:** Chao Feng, Jing Chen, Wenxin Ye, Kaisen Liao, Zhanshi Wang, Xiaofei Song, Mingqiang Qiao

**Affiliations:** ^1^ Department of Urology, Tongde Hospital of Zhejiang Province, Hangzhou, China; ^2^ The Key Laboratory of Molecular Microbiology and Technology, Ministry of Education, College of Life Sciences, Nankai University, Tianjin, China; ^3^ The Key Laboratory of Bioorganic Synthesis of Zhejiang Province, College of Biotechnology and Bioengineering, Zhejiang University of Technology, Hangzhou, China; ^4^ College of Life Sciences, Shanxi University, Taiyuan, China

**Keywords:** resveratrol, physiological function, microorganism, metabolic engineering, synthetic biology

## Abstract

Resveratrol, a bioactive natural product found in many plants, is a secondary metabolite and has attracted much attention in the medicine and health care products fields due to its remarkable biological activities including anti-cancer, anti-oxidation, anti-aging, anti-inflammation, neuroprotection and anti-glycation. However, traditional chemical synthesis and plant extraction methods are impractical for industrial resveratrol production because of low yield, toxic chemical solvents and environmental pollution during the production process. Recently, the biosynthesis of resveratrol by constructing microbial cell factories has attracted much attention, because it provides a safe and efficient route for the resveratrol production. This review discusses the physiological functions and market applications of resveratrol. In addition, recent significant biotechnology advances in resveratrol biosynthesis are systematically summarized. Furthermore, we discuss the current challenges and future prospects for strain development for large-scale resveratrol production at an industrial level.

## Introduction

**FIGURE 1:**
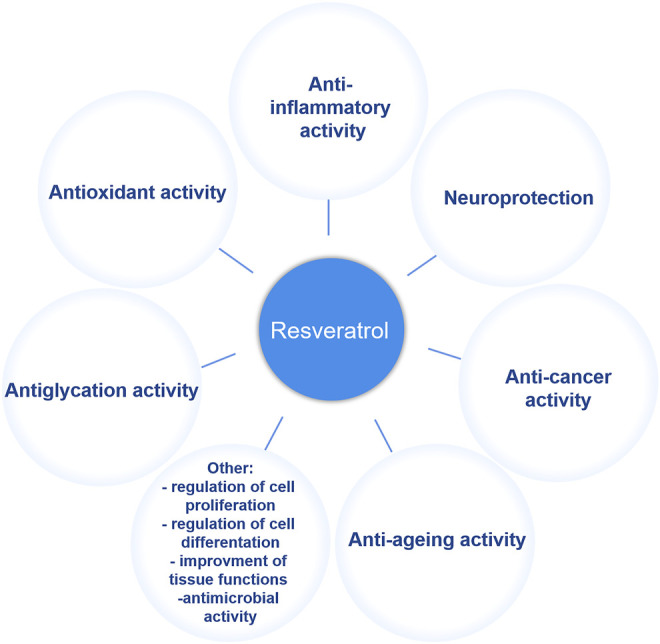
Physiological function of resveratrol.

Resveratrol has been universally found in a variety of plants including berries, bilberries, peanuts, grapes and even ferns since it was initially extracted from the root of white hellebore (Veratrum grandiflorum) in 1940 (Lim et al., 2011; Jeandet et al., 2012; Li et al., 2015). In plants, resveratrol, the *de novo* synthetic phytoalexin, acts as a protector against pathogen invasion and infection (Tian and Liu, 2020). It is known that two isomeric forms exist in nature, including *cis-* and *trans*-resveratrol, but the *trans* isomer is the primary biologically-active form. The anti-tumor properties of resveratrol in multiple human organs or systems, include breast (Sinha et al., 2016), cervical (Liu Q et al., 2020), uterine (Sexton et al., 2006), blood (Breuss et al., 2019), kidney (Den Hartogh and Tsiani, 2019), liver (Jakubczyk et al., 2020), eye (Bola et al., 2014), bladder (Almeida and Silva, 2021), thyroid (Giuliani et al., 2017), esophageal (Zhou et al., 2003), prostate (Zaffaroni and Beretta, 2021), brain (Kiskova et al., 2020), lung (Feng et al., 2016), skin (Ravikumar et al., 2019), gastric (Zulueta et al., 2015), colon (Li et al., 2019), head and neck (Shrotriya et al., 2015), bone (Chen et al., 2019), ovarian (Sirotkin et al., 2020), and cervical (Sun et al., 2021), and have been extensively studied over the last few decades (Baur and Sinclair, 2006). Moreover, as a plant secondary metabolite, resveratrol has also been noted for many pharmacological applications such as an anti-oxidant, anti-inflammatory, anti-aging, and neuroprotective agent, as well as for many other properties (Rauf et al., 2018) (Figure 1). However, the effects of resveratrol, especially its clinical effects on human health, currently must be further verified and studied because of a limited number of human studies and small cohort sizes. Nevertheless, considering its various physiological activities, resveratrol has attracted much attention in the pharmaceutical, cosmetic and many other industries. Because of an increasing demand in all walks of life, large-scale resveratrol production is urgently needed.

It is difficult to meet the current industry demand for resveratrol by extracting and purifying it from plants, due to the complex process, high production cost and low yield (Sáez-Sáez et al., 2020). Although resveratrol production can currently be increased by employing chemical synthesis, the complex production process, the requirement for toxic solvents and the production of byproducts limits large-scale production (Shrestha et al., 2019). It is noteworthy that the use of microorganisms has made significant contributions to the biosynthesis of pharmaceutical and industrial compounds in recent decades, because of its low production cost, high efficiency, high product purity and simple genetic operation process, and it is considered to have a promising potential for the production of natural products (Cravens et al., 2019). *De novo* resveratrol biosynthesis via metabolic engineering and synthetic biology in microorganisms provides a feasible way to produce resveratrol, and this has attracted worldwide interest (Liu et al., 2019; He et al., 2020; Liu Z et al., 2020; Yuan et al., 2020; Costa et al., 2021). This review discusses the current status and progress of resveratrol production in recent years, as well as optimization strategies for related hosts, pathways and enzymes for resveratrol production. Hopefully in the next few years, researchers will continue to improve process engineering strategies, and increasingly utilize metabolic and protein engineering to meet a series of more complex biosynthetic challenges.

## Microorganism Hosts for Resveratrol Production

Many properties should be considered when selecting hosts for natural products production. Due to a long history of research, microorganisms have been widely chosen because of mature techniques such as gene editing and large-scale fermentation, particularly for metabolic engineering (Yang et al., 2021). With the increasing demand for resveratrol, many studies have focused on heterologous resveratrol production in prokaryotes such as *Escherichia coli*, *Corynebacterium glutamicum* and *Streptomyces venezuelae*, and in eukaryotes, including *Saccharomyces cerevisiae* and *Yarrowia lipolytica* (Dudnik et al., 2018; Cravens et al., 2019). Table 1 presents studies using metabolically engineered strains to produce resveratrol.

**TABLE 1:** Biosynthesis of resveratrol in engineered microorganisms.

Microbial host	Pathway genes (source)	Pathway/Host engineering	Substrate	Titer (mg/L)	References
*S. cerevisiae* W303-1A	4CL1 (*A. thaliana*)	PAD1 knockout	*p*-Coumaric acid	3.1	Shin et al. (2011)
	STS (*A. hypogaea*)				
*S. cerevisiae* WAT11	TAL (*R. sphaeroides*)	Expression of araE transporter (*E. coli*)	Tyrosine	3.1	Wang et al. (2011)
	4CL:STS, 4CL1 (*A. thaliana*)-STS		*p*-Coumaric acid	2.3	
	*(V.vinifera*) fusion enzyme		Grape Juice	3.44	
*S. cerevisiae* W303-1A	PAL (*R. toruloides*)	Overexpression of ACC1	Tyrosine	5.8	Shin et al. (2012)
	C4H, 4CL1 (*A. thaliana*)				
	STS (*A. hypogaea*)				
*S. cerevisiae* WAT11	4CL1 (*A. thaliana*)	Synthetic scaffold	*p*-Coumaric acid	14.4	Wang and Yu, (2012)
	STS (*V. vinifera*)				
*S. cerevisiae* WAT11	4CL:STS, 4CL1 (*A. thaliana*)-STS	Overexpression of AAE13	*p*-Coumaric acid	Up to 3.7	Wang et al. (2014)
	*(V. vinifera*) fusion enzyme				
*S. cerevisiae* EC1118	4CL (*A. thaliana*)	—	*p*-coumaric acid	8.249	Sun et al. (2015)
	STS (*V. vinifera*)				
*S. cerevisiae* CEN.PK102-5B	TAL (*H. aurantiacus*) TAL (*F. johnsoniae*) 4CL1 and 4CL2 (*A. thaliana*)	Overexpression of ARO4fbr,ARO7fbr, and ACC1	Glucose (Fed-batch)	415.65	Li et al. (2015)
	RS (*V. vinifera*)		Ethanol (Fed-batch)	531.41	
*S. cerevisiae* CEN.PK102-5B	PAL2, C4H, 4CL2 (*A. thaliana*)	Overexpression of ARO4fbr, ARO7fbr, ACC1, CYB5 (*S.cerevisiae*), ATR2 *A. thaliana*), ACS (*S. enterica*), and deletion of aro10	Glucose (Fed-batch)	812	Li et al. (2016)
	VST1 (*V. vinifera*)		Ethanol (Fed-batch)	755	
*Y. lipolytica*	4CL (*N. tabacum*)	Overexpression of:ACC1, PEX10	*p*-Coumaric acid	48.7	Palmer et al. (2020)
	STS (*A. hypogaea*)				
*Y. lipolytica* Po1d (wt), derived from W29	TAL (*F. johnsoniae*)	—	Glycerol	430	He et al. (2020)
	PAL (*V. vinifera*)				
	C4H, 4CL1 (*A. thaliana*)				
	VST (*V. vinifera*)				
*Y. lipolytica* ST6512 (W29)	TAL (*F. johnsoniae*)	Overexpression of:ARO4fbr and ARO7fbr	Glucose	409	Sáez-Sáez et al. (2020)
	4CL1 (*A. thaliana*) VST1 (*V. vinifera*)		Glucose (Fed-batch)	12,355	
*C. glutamicum* DelAro3	STS (*A. hypogaea*)	Deletion of *phdB, pcaF* and *pobA*	*p*-Coumaric acid	12	Kallscheuer et al. (2016)
	4CL (*P. crispum*)		*p*-coumaric acid +	158	
			cerulenin		
*C. glutamicum* DelAro4	TAL (*F. johnsoniae*) 4CL (*Petroselinum*) STS (*A. hypogaea*) *aroH* (*E. coli*)	Deletion of *phdB, pcaF, qsuB* and *pobA*	Glucose	12	Braga et al. (2018b)
			Glucose + cerulenin	59	
			Glucose (40 g/L)	4	
			Glucose (80 g/L)	12	
			Glucose (Fed-batch)	7	
*E.coli* BW27784	4CL (*A. thaliana*)	—	*p*-Coumaric acid	404	Lim et al. (2011)
	STS (*A. hypogaea*)				
	4CL (*A. thaliana*)			1,380	
	STS (*V. vinífera*)				
	4CL (*P. crispum*)			142	
	STS (*A. hypogaea*)				
	4CL (*P. crispum*)			610	
	STS (*V. vinífera*)				
	4CL (*A. thaliana*)			2,340	
	STS (*V. vinífera*)				
*E. coli* C41 (DE3)	TAL (*Saccharothrix espanaensis*)	—	*p*-Coumaric acid	1.4	Choi et al. (2011)
	4CL (*Streptomyces coelicolor*)				
*E. coli* BL21 (DE3)	TAL (*R. glutinis*)	—	Tyrosine	35.02	Wu et al. (2013)
	4CL (*P. crispum*)				
	STS (*V. vinifera*) *matB* and *matC* (*R. trifolii*)				
*E. coli* C41 (DE3)	TAL (*S. espanaensis*)	—	Glucose	5.2	Kang et al. (2014)
	4-CL (*S. coelicolor*)				
	STS (*A. hypogaea*)				
*E. coli* BW27784	4CL (*A. thaliana*)	—	*p*-Coumaric acid	160	Afonso et al. (2015)
	STS (*A. hypogaea*)				
*E. coli* BL21 (DE3)	TAL (*S. espanaensis*)	—	Tyrosine	114.2	Wang et al. (2015)
	4-CL (*A. thaliana*)				
	STS (*A. hypogaea*)				
*E. coli* BW25113	4CL2 (*P.crispum*)	—	*p*-Coumaric acid	268.2	Yang et al. (2015)
	STS (*V. vinifera*)				
*E. coli* BW25113 (DE3)	TAL (*R. glutinis*)	Inactivation of *tyr*R and deletion of *trp*ED by chromosomal integration	Glucose	4.6	Liu et al. (2016)
	4CL (*P. crispum*)				
	STS (*V. vinifera*)				
*E.coli* W (pheA-) Rg	TAL (*R. glutinis*) *tktAfbr* and *aroGfbr* (*E. coli*)	Deletion of *phe*A	Glycerol	22.58	Camacho-Zaragoza et al. (2016)
*E.coli* W-Vv	4CL (*S. coelicolor*) STS (*V. vinífera*)				
*E. coli* BL21 (DE3)	TAL (*Trichosporon cutaneum*)	Down-regulation of *fabD, fabH, fabB*, f*abF, fabI*	Glucose	304.5	Wu et al. (2017)
	4CL (*P. crispum*)				
	STS (*V. vinifera*) *matB* and *matC* (*R. trifolii*) *tyrAfbr* and *aroGfbr* (*E.coli K12*)				

## Yeast Hosts

Yeasts are considered as a more suitable platform than bacteria for the expression of natural products, because of their ability to carry out eukaryotic post-translational modifications and feasibility of genetic manipulation (Madzak, 2015; Braga et al., 2018a). Yeasts and plants have a similar endoplasmic reticulum, an intracellular compartment to support eukaryotic and membrane protein biosynthesis (Rainha et al., 2020). *S. cerevisiae* is a Generally Regarded As Safe (GRAS) organism that is widely used for pharmaceutical products and food markets because of its safety, and it is more commonly used for resveratrol production than other host species (Fletcher et al., 2016; Pereira et al., 2019). Becker et al. (2003) reconstructed for the first time a biochemical pathway in a microorganism to produce resveratrol and obtained a titer of 0.00145 mg/L resveratrol in yeast. The biosynthesis of resveratrol via a tyrosine intermediate has been achieved in *S. cerevisiae*; this was a first time demonstration of the possibility of *de novo* resveratrol biosynthesis from glucose. By using a pull-push-block strain engineering strategy, 800 mg/L resveratrol was produced by the engineered host strains (Li et al., 2015; Li et al., 2016). Yuan et al. (2020) constructed a consortium system for *de novo* resveratrol biosynthesis and obtained 36 mg/L resveratrol. *Y. lipolytica*, another yeast, has been widely concentrated in industrial area for more than 50 years because of its high production capacity for organic acids, which are widely used in diverse research areas (Ma et al., 2020) (Madzak, 2018). Gu et al. (2020) created resveratrol-producing strains of *Y. lipolytica*, in which could produce 12.67 ± 2.23 mg/L of resveratrol with glucose as the substrate. He et al. (2020) engineered *Y. lipolytica* as a vehicle for high-level resveratrol production and obtained 0.43 g/L resveratrol by exploiting the tyrosine and the phenylalanine branches of the pathway. Recently, Sáez-Sáez et al. (2020) tried to improve the resveratrol titer in *Y. lipolytica* by metabolic engineering, resulting in 12.4 ± 0.3 g/L resveratrol, which is the highest titer for *de novo* resveratrol production up to now.

## Bacterial Hosts

*E. coli* has been the subject of industrial interest for resveratrol production because of its fast growth the availability of advanced technology for its genetic manipulation and synthetic biology (Braga et al., 2018a). Furthermore, tyrosine and *p*-coumaric acid, the basic precursors of resveratrol, are the critical for increasing production, and are easily assessable and improved in *E. coli via* multiple metabolic engineering strategies (Shrestha et al., 2019). Additionally, *E. coli* is more suitable for resveratrol production than yeast because of its high tolerance to *p*-coumaric acid, another advantage (Shin et al., 2011; Huang et al., 2013) (Donnez et al., 2009). Recently, Zhang et al. identified stilbene synthase as the limiting enzyme via a novel probabilistic computational model and improved the final resveratrol titer from 62.472 mg/L to 172.799 mg/L, proving the model useful for predicting and improving biological production (Cotner et al., 2021).

Engineered *C. glutamicum* has also been employed as a vehicle for resveratrol production. Kallscheuer et al. (2016) introduced TAL from *Flavobacterium johnsoniae* into a strain of *C. glutamicum* for resveratrol production; 60 mg/L resveratrol was produced when using glucose as the carbon source. They further achieved 5 mg/L *trans*-resveratrol in *C. glutamicum* from 4-hydroxybenzoate, which is the first time a phenylpropanoid was synthesized from 4-hydroxybenzoic acid other than aromatic amino acids and ammonia lyase (Kallscheuer et al., 2017). Milke et al. (2019) constructed a recombinant *C. glutamicum* stain and increased the titer of resveratrol to 112 mg/L by modulating the central carbon metabolism of the host strain.

Engineered *S. venezuelae* has also been employed to produce resveratrol. Park and others reported resveratrol synthesis by expressing the heterologous phenylpropanoid biosynthetic pathway genes in *S. venezuelae* for the first time, although they only obtained 0.4 mg/L of resveratrol (Park et al., 2009). Likewise, the use of other organisms such as *Lactobacillus lactis* and *Aspergillus niger* has also been reported for resveratrol bioproduction (Chong et al., 2012).

## Metabolic Engineering to Enhance Resveratrol Production

**FIGURE 2:**
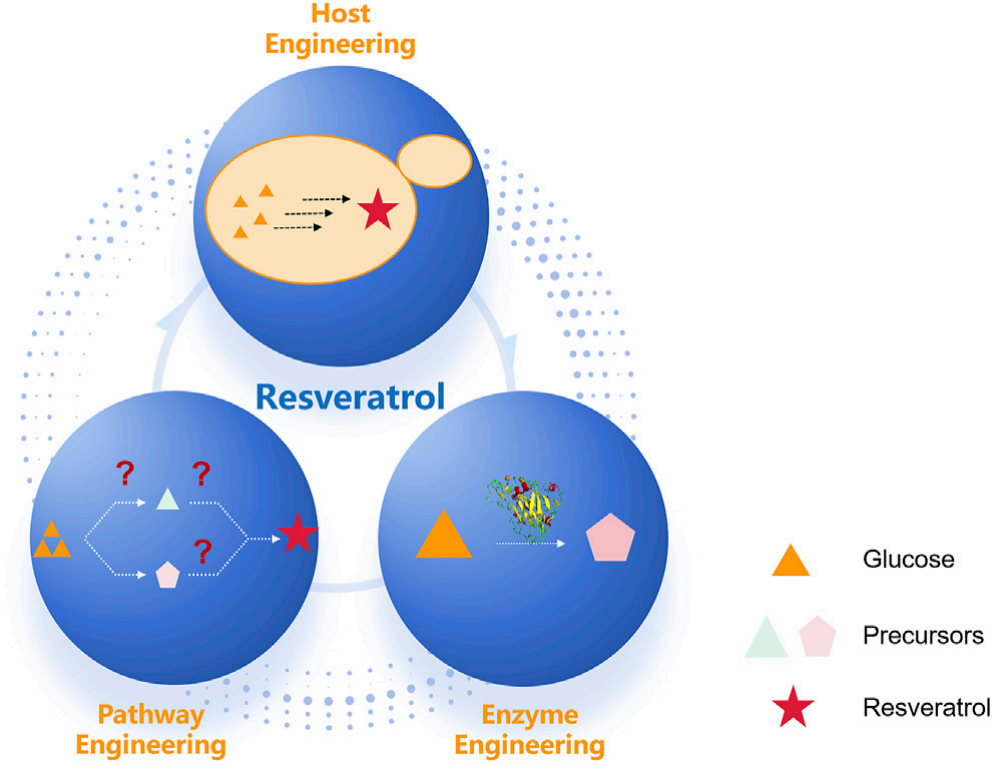
Metabolic engineering at multiple levels has enabled engineering of increasingly complex heterologous resveratrol pathways. Heterologous resveratrol production in a microbial host can involve engineering at three different scales: host, pathway, and enzyme.

The design-build-test cycle (DBT cycle) has been widely used in metabolic engineering for the production of plant natural products (Nielsen and Keasling, 2016). In DBT iterative engineering cycles, host engineering includes the sufficient provision of precursor via precursor metabolites overproduction. Pathway engineering includes biosynthesis via a heterologous route to produce natural products, and enzyme engineering includes directed evolution to improve the properties of key rate-limiting enzymes (Cravens et al., 2019) (Figure 2). The rapid development of synthetic biology and enabling technology has accelerated DBT iterative engineering cycles, which have been widely employed for the engineering of resveratrol biosynthesis.

## Host Engineering

In recent years, significant progress toward high-level resveratrol production has been achieved by microbial metabolic engineering. Nevertheless, an insufficient precursor supply (i.e., aromatic amino acids and malonyl-CoA) is still the main rate-limiting factor for resveratrol production in heterologous hosts. Thus, increasing the precursor supply via genetic manipulation of the host strain is considered a fundamental strategy for resveratrol bioproduction (van Summeren-Wesenhagen and Marienhagen, 2013; Milke et al., 2018).

**FIGURE 3:**
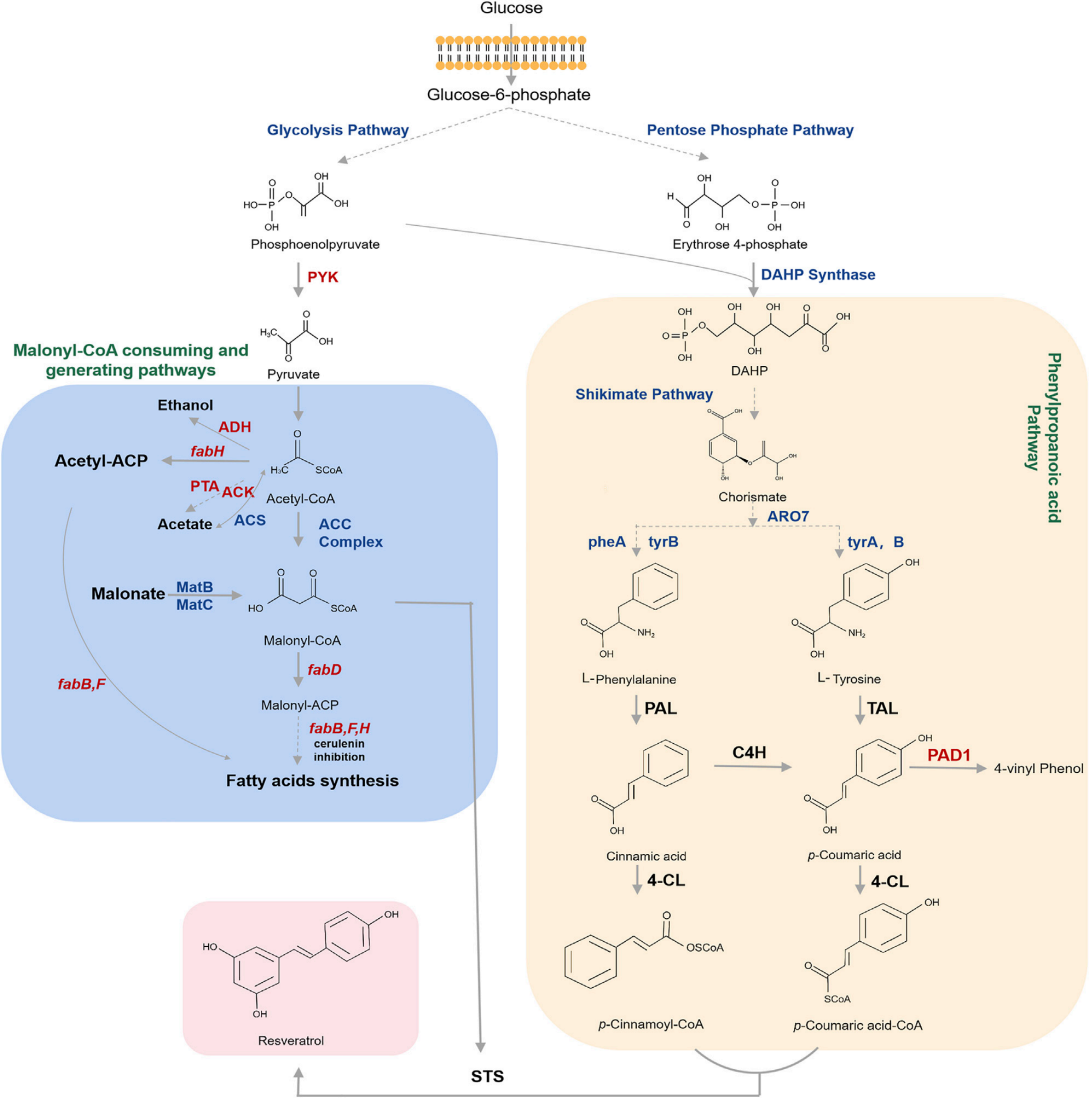
Biosynthetic pathway for resveratrol starting from glucose. The pathways for generating precursors of resveratrol biosynthesis such as phenylpropanoyl-CoAs and malonyl-CoA are highlighted. Dotted arrows refer to multiple steps. Genes and enzymes in blue are targets for overexpression. Genes and enzymes in red are targets for knockout or inhibition. Note that the malonate is externally supplied. DAHP Synthase, 3-deoxy- d-arabinoheptulosonate-7-phosphate (DAHP) synthase; DAHP, 3-deoxy-D arabinoheptulosonate 7- phosphate; ARO7, chorismate mutase; ARO7, chorismate mutase; tyrA/pheA, genes that encode the chorismate mutase protein; tyrB, gene that encodes the tyrosine aminotransferase; PAL, phenylalanine ammonia lyase; TAL, tyrosine ammonia-lyase; C4H, cinnamate 4-hydroxylase; PAD, phenyl acrylic acid decarboxylase; 4CL, 4-coumaroyl-coA ligase; STS, stilbene synthase; PYK, pyruvate kinase; Acetyl-CoA, acetyl-coenzyme A; ADH, alcohol dehydrogenases; fabH, gene that encodes 3-oxoacyl carrier protein synthase III; PTA, phosphate acetyltransferase; ACK, acetate kinase; ACS: acetyl-CoA synthase; Acetyl-ACP, acetyl-acyl carrier protein; ACC complex, acetyl-coA carboxylase multienzyme complex; Malonyl-CoA, malonylcoenzyme A; MatB, malonyl-CoA synthetase; MatC, malonate carrier protein; fabD, gene that encodes the malonyl-CoA-acyl carrier protein transacylase; Malonyl-ACP, malonyl-acyl carrier protein; fabB/fabF, genes that encode the beta-ketoacyl-acp synthase I/II protein.

As shown in Figure 3, resveratrol is produced from the aromatic amino acids l-phenylalanine (L-Phe) or l-tyrosine (L-Tyr). The metabolic engineering of the shikimic acid pathway primarily regulates the carbon flux into chorismate, followed by L-Phe and L-Tyr (Jiang et al., 2005; Rodriguez et al., 2015). Frequently-used strategies to increase the biosynthetic flux of the shikimic acid pathway are the elimination of enzyme feedback inhibition and the regulation of transcription. Furthermore, extending the supply and availability of erythrose-4-phosphate (E4P) and phosphoenolpyruvate (PEP) are the primary methods for improving chorismate production (Bulter et al., 2003; Lütke-Eversloh and Stephanopoulos, 2007). Significant strategies have been developed to enhance the production of aromatic amino acids or derived phenylpropanoic acids in microorganisms (Juminaga et al., 2012; Zhang and Stephanopoulos, 2013; Rodriguez et al., 2015). For example, Juminaga et al. (2012) reported the biosynthesis of resveratrol by encoding the key enzymes for converting E4P and PEP to L-Tyr, which significantly increased L-Tyr production to 80% of the theoretical yield.

Malonyl-CoA also serves as an important precursor for resveratrol biosynthesis. Moreover, malonyl-CoA is mostly used as an essential intermediate for fatty acid biosynthesis to support cell growth, so only a limited level of malonyl-CoA remains for resveratrol biosynthesis, which is a major challenge in resveratrol production. Therefore, two prime strategic steps have been utilized in order to expand the intracellular malonyl-CoA pool in microorganisms: 1) repressing fatty acid biosynthesis to inhibit malonyl-CoA consumption; 2) expanding the cytoplasmic malonyl-CoA pool by carboxylation of acetyl-CoA carboxylase (ACC), which can increase the amount of acetyl-CoA carboxylation to malonyl-CoA. Zha et al. (2009) found that overexpression of ACC increased the concentration of malonyl-CoA in *E. coli*. In addition, deleting genes encoding competing pathways, such as *pta* and *ackA,* which are involved in the degradation of acetyl-CoA into acetic acid, and the *adhE* gene, which is involved in ethanol production, can achieve significant effects (Finzel et al., 2015). Yang et al. (2015) revealed that blocking malonyl-CoA consumption and the deletion of the *fab* genes were lethal in microorganisms. Thus, three procedures have been carried out to inhibit malonyl-CoA consumption: 1) inhibiting FabB and FabF by adding the antibiotic cerulenin (Lim et al., 2011; Lu et al., 2016); 2) using antisense RNA to repress the *fab* operon, especially the *fabD* genes (Wu et al., 2014; Yang et al., 2015); 3) using CRISPRi technology to inhibit the *fab* gene and direct the carbon flux toward malonyl-CoA (Wu et al., 2015; Liang et al., 2016). Furthermore, another significant approach is to introduce biosynthetic pathway genes for malonate assimilation, such as *matB* and *matC*, into the system. (Shin et al., 2011).

## Pathway Engineering

By introducing the entire biosynthetic pathway into microorganisms, efficient synthesis of resveratrol from precursors (L-Phe or L-Tyr) or low-cost materials (such as glucose, ethanol or glycerol) can be realized, which is a great help for *de novo* biosynthesis or biotransformation of resveratrol (Jeandet et al., 2012; Li et al., 2016; Chen et al., 2020). The biosynthetic pathway for resveratrol is shown in Figure 3. The secondary metabolite phenylpropanoid route is the major metabolic pathway for resveratrol biosynthesis (Lu et al., 2016). The first step in resveratrol biosynthesis is the production of phenylpropanoic acids (i.e., *p*-coumaric acid and cinnamic acid) through nonoxidative deamination via tyrosine ammonia lyase (TAL) and l-phenylalanine ammonia lyase (PAL), which are then converted to *p*-coumaroyl-CoA and cinnamoyl-CoA by 4-coumarate-CoA ligase (4CL). Cinnamic acid can also be hydroxylated with the assistance of cinnamic acid-4-hydroxylase (C4H) to form *p*-coumaric acid. Finally, malonyl-CoA is condensed with *p*-coumaroyl-CoA to produce resveratrol, catalyzed by stilbene synthases (STSs) (van Summeren-Wesenhagen and Marienhagen, 2015; Milke et al., 2018). The main objective for the engineering of this pathway is to efficiently convert the aromatic amino acids to phenylpropyl by introducing hyper-active ammonia lyases such as PAL or TAL (Huang et al., 2013; Zhang and Stephanopoulos, 2013), which is a bottleneck in the resveratrol production from glucose (Yang et al., 2015; Kallscheuer et al., 2016). Liu et al. (2016) introduced TAL, 4CL and STS genes into *E. coli* strain and obtained 4.6 mg/L of resveratrol from glucose. Soon after, Wu et al. (2017) applied multiple metabolic engineering approaches to produce resveratrol from glucose in *E. coli*. However, the low activity of TAL and PAL enzymes is still the main obstacle for introducing the entire *de novo* pathway into the microorganism.

## Enzyme Engineering

As mentioned above, in many cases the function of one or a few key enzymes acts as a bottleneck in the overall metabolic fluxes, which are considered as the rate-limiting steps. Microorganisms cannot produce enough targeted product because of enzymes that have limited turnover or poor expression (Song et al., 2017; Song et al., 2018; Lian et al., 2018). Therefore, protein engineering, particularly directed evolution, has become one of the most powerful and widespread tools for engineering improved or novel functions in enzymes (Wu et al., 2013; Wang et al., 2021). Researchers employed protein engineering of 4CL and STS in *E. coli* for higher and more efficient resveratrol production (Becker et al., 2003; Zhang et al., 2015). Likewise, a yeast host harboring codon-optimized TAL and fused 4CL and STS, which allowed the production of 1.06 mg/L resveratrol without the use of L-Tyr (Wang et al., 2011). Moreover, the robustness of the rate-limiting enzymes or the metabolic activity of pathways can be optimized *in vivo* via metabolite-responsive biosensors (Skjoedt et al., 2016). Xiong et al. (2017) selected resveratrol hyper-producers rapidly and efficiently by reapplying the TtgR regulatory protein to a resveratrol-responsive biosensor in *E. coli*. Compared to the wild type, 4CL variants displayed improved catalytic properties for the production of this aromatic compound.

## Production Process Optimization to Increase Resveratrol Synthesis

The balance and optimization of microbial growth and product formation have been identified as essential for increasing resveratrol production. In order to satisfy the world’s sustainable demand, researchers have conducted some important studies for large-scale industrial production. Braga et al. (2018b) observed that increasing the glucose concentration from 40 g/L to 80 g/L resulted in a resveratrol titer that increased from 4 mg/L to 12 mg/L, which demonstrated that proper culture conditions, i.e., substrate concentration, were essential for the resveratrol production in *C. glutamicum* (Braga et al., 2018b).

In order to optimize and construct recombinant strains, the metabolic burden caused by the competition between natural metabolism and chemical production pathways (including chemical precursors, energy molecules and reduction equivalents) is one of the most important challenges to be urgently faced and resolved. Considering that polyphenols such as resveratrol are produced through complex biosynthetic pathways, the concept of co-culture has gained increasing attention in recent years. Through co-culture, that is, using multiple strains to produce different products or metabolize different substrates, it is feasible to co-produce resveratrol. Furthermore, the entire pathway can be divided and introduced into each strain as an entire module (Zhou et al., 2015). Yuan et al. (2020) recently described an approach utilizing a co-culture of *E. coli*–*S. cerevisiae* to produce resveratrol, with a final titer of 36 mg/L using glucose as a carbon source. To alleviate the metabolic burden of a single host, researchers divided the labor among artificial microbial communities using a cell consortium strategy (Yuan et al., 2020).

There are many other obstacles to resveratrol production in microbial hosts, such as the high cost of precursors and precursor toxicity. Researchers recently attempted to overcome these difficulties by using engineered strains to obtain low-cost and sustainable substrates and by using fed-batch cultures to reduce the toxicity of precursors (Wu et al., 2017) (Watts et al., 2006; Huang et al., 2013; Zhang and Stephanopoulos, 2013).

## Conclusion and Future Perspectives

As of now, the efforts and results mentioned above have demonstrated the feasibility of converting microorganism hosts into cell factories to produce resveratrol, which can be achieved by grafting exogenous biosynthetic pathways into the endogenous metabolic network of cells. However, the current problem is that although they have promising potential for development and popularization, at present the performance of most engineered strains cannot achieve the goals required for industrial production. How to use microorganisms as vehicles to produce resveratrol more economically is still an important challenge. Multiple factors, including the cytotoxicity of end products, the low activity and stability of catalytic enzymes, and metabolic imbalances at the biosynthetic pathway level and across the cellular network, are challenges for the advancement of cell growth, the rate of production, product titer and yield to a certain degree. Furthermore, the production process is also hampered by the lack of critical, basic information about the interactions and regulation of metabolic networks, which require more time to discover and verify the contribution of potential biosynthetic pathways for resveratrol production. However, with the recent rapid development of metabolic engineering principles, it is expected that novel and reliable solutions will be found that will break the shackles that hinder industrial biological resveratrol production.

The cellular adaptation and metabolic stability of engineered cell factories has frequently been affected by heterologous chemicals, which are tremendously cytotoxic during the process of biosynthesis and long-term accumulation. Consequently, some approaches, including adaptive laboratory evolution (ALE) (Sandberg et al., 2019) and the multi-functional genome-wide CRISPR (MAGIC) system (Lian et al., 2019), have been designed to reduce the toxicity of products while maximizing the potency and yield of chemical products. For instance, Pereira et al. (2019) recently investigated through ALE experiments the mechanism of tolerance of *S. cerevisiae* to the cellular stress imposed by inhibiting concentrations of dicarboxylic acids. Lian et al. (2019) improved furfural tolerance in yeast using the MAGIC system; a method that identifies complex phenotypic genetic determinants that have not been previously identified, especially those interacting synergistically when disturbed to different levels of expression. The novel strategies mentioned above represent a promising alternative strategy to improve the resveratrol production capability of microbial hosts.

In order to alleviate the rate-limiting steps, protein engineering, especially directed evolution, has been applied to improve enzyme properties. It is particularly worth noting that machine learning has been increasingly utilized for protein engineering. Luo et al. developed a high-performance method called Evolutionary Context-Integrated Neural Network (ECNet), providing generalization from low-order mutants to higher-order mutants, which can predict protein function levels from sequence to protein engineering process (Luo et al., 2021). Besides, recent advances in the development of sequence-based, MD-based, structure-based, and machine learning-based computational tools will promote the identification of the beneficial mutations and accelerate the protein engineering process by creating smaller but smarter libraries to enhance the robustness of catalytic enzymes (Huang et al., 2016). Moreover, attention should be paid to a combinatorial method to guide every precursor and metabolite towards the large-scale resveratrol production. Additionally, for resveratrol biosynthesis processes based on a microbial platform, the application of complete biosynthetic and related knowledge of molecular biology, including the entire genome, transcriptome, proteome and metabolome, will be promising for the improvement of the production efficiency and yield of resveratrol.

In summary, the goal of efficient resveratrol production in microbial hosts can be advanced by the integration of multiple tools such as metabolic engineering, systems and synthetic biology for strain design, as well as by improving process engineering strategies. By using such strategies, heterologous resveratrol production can be competitive with existing chemical synthesis and plant extraction processes, which will be a better choice to achieve the goal of sustainable resveratrol production.
